# Evaluation of Food Safety Knowledge and Attitudes Among Adults in the United Arab Emirates

**DOI:** 10.7759/cureus.54451

**Published:** 2024-02-19

**Authors:** Zainab Z Alkhamis, Hana M Musthafa, Mohammed Ali Al-Hamadani, Anusha Sreejith, Syed Zain Ali

**Affiliations:** 1 Internal Medicine, Gulf Medical University, Ajman, ARE; 2 Community and Family Medicine, Gulf Medical University, Ajman, ARE; 3 Community Medicine/Demography, Gulf Medical University, Ajman, ARE

**Keywords:** foodborne diseases, foodborne pathogens, food safety regulations, food safety and hygiene, food handling

## Abstract

Background

Food is handled by many individuals in large food setups, therefore increasing the chance of contamination that leads to foodborne diseases (FBDs). This study was purposed to evaluate adults' understanding of food safety, FBDs, and hygiene practices across various demographic groups in the United Arab Emirates (UAE) and to explore the link between their knowledge of food safety and their corresponding attitudes.

Methods

A cross-sectional study was conducted with 402 adults using a validated, self-administered questionnaire available in both printed and online formats. The study was carried out at Gulf Medical University and Thumbay hospitals and clinics over six months, beginning in December 2022 to June 2023. Data analysis was performed using IBM SPSS Statistics for Windows, Version 26.0 (Released 2019; IBM Corp., Armonk, New York, United States). The chi-squared test was employed to examine the association between variables, and significant associations were further analyzed through logistic regression.

Results

Out of the 402 participants, the population was predominantly female 275 (67.9%), and from Southeast Asia 222 (55.4%), with students comprising the largest occupational group 186 (47%). Only 106 (26.36%) had received food safety training, and a mere 187 (46.51%) demonstrated adequate knowledge. Awareness levels varied, with the highest for raw food safety (64.02%) and the lowest for canned foods (40.79%). Demographic analysis revealed significant associations: males exhibited more inadequate knowledge 79 (62.2%) than females 136 (49.4%), and students showed higher inadequacy 104 (55.9%) compared to healthcare workers 31 (35.6%). Positive attitudes towards food safety were prevalent 226 (56.2%), and positive attitudes were found in women 157 (57.1%), individuals above 30 years of age 110 (50.5%), individuals working in healthcare 140 (62%), and married individuals 117 (60.9%). With a strong correlation (p<0.001), women were 1.68 times more likely to possess adequate knowledge than men (95% CI: 1.09, 2.59), and healthcare workers were 2.33 times more likely than students (95% CI: 1.37, 3.95).

Conclusion

The study reveals a low level of knowledge about food safety among adults in the UAE. Therefore, emphasis should be placed on increasing awareness of these concepts to reduce the burden of FBDs on the healthcare system.

## Introduction

Foodborne diseases (FBDs) remain prevalent as a global public health concern, even in regions with well-developed food safety systems [1]. According to reports from the Food and Agriculture Organization (FAO) and World Health Organization (WHO), unsafe food intake affects millions of people worldwide [2,3]. The WHO estimates the global cases of food poisoning at approximately 600 million annually, with 420,000 resulting in deaths due to FBDs [3]. These diseases stem from the ingestion of contaminated food with microorganisms or toxins, often leading to mild to severe clinical symptoms and, in advanced cases, death [4]. FBDs are particularly severe in developing countries due to weak regulatory systems, inadequate food safety laws, and poor sanitation [5]. Notably, the majority of food poisoning cases originate at home, emphasizing the crucial role of domestic food handlers in the food supply chain [6].

Food safety, also known as food hygiene, refers to measures taken to prioritize food safety throughout the supply chain, with primary responsibility lying with food handlers. Food handlers encompass individuals involved in various aspects of food preparation, including harvesting, slaughtering, storage, transportation, processing, and preparation, and their knowledge and practices during food handling significantly impact food hygiene. Contamination can occur at any point in the food production chain, underscoring the importance of safe and hygienic food handling practices by food handlers to prevent the spread of FBDs. Studies have highlighted that poor food safety knowledge and unsafe practices among food handlers are associated with the highest risk of FBD transmission [5,7].

Adults, often the primary food handlers in their families or for themselves, pose a significant risk for FBD outbreaks in domestic settings [8]. Adults with inadequate knowledge of safe food handling practices may engage in risky food preparation practices, leading to food contamination [9]. Additionally, attitudes towards food safety play a crucial role in translating knowledge into observable practices. Therefore, assessing the food safety knowledge and attitudes of adult food handlers is paramount. This study aims to examine the comprehension, feelings, and adherence to food safety regulations among food handlers in the United Arab Emirates (UAE), providing valuable baseline data for food establishments to create, implement, and uphold efficient food safety protocols.

## Materials and methods

This cross-sectional study was conducted among adults residing in the UAE to evaluate the level of knowledge of food safety and hygiene practices within healthcare sector partners in the UAE and to establish the correlation between comprehension of food safety. The study took place at Gulf Medical University and Thumbay hospitals and clinics from December 2022 to June 2023.

The inclusion criteria encompassed all adults aged 18 years or above who were working in public food setups in the UAE or in direct contact with food preparation and handling at the time of data collection. Exclusion criteria included adults unwilling to participate or those living outside of the UAE.

The sample size was determined through a thorough literature review, using the following single population formula: \(\{4pq}{L^2^}=\frac{(4×0.5×0.5}{(0.05)^2^})=400\).

The prevalence of adequate knowledge of food safety in previous studies was assumed to be 50%. Accounting for a 10% non-response rate, the sample size was calculated as follows: sample size=400.

Data collection instrument and sampling procedure

A self-administered questionnaire (see Appendices) was formulated after conducting a thorough review of the literature on similar studies and was subsequently validated by three experts in the field [6-9]. The research was conducted ethically, ensuring confidentiality and anonymity, and informed consent was obtained from all participants. Permission for the study was granted by the Institutional Review Board of Gulf Medical University (approval number: 1) before initiating the research. Following ethical approval from the IRB, permission was received from hospitals and universities to conduct the research.

The questionnaire comprised three sections, with the first focusing on collecting sociodemographic details of the participants, including any formal food safety training they had received and the method through which they acquired knowledge of food safety methods. The second section encompassed 25 questions divided into five subsections, aimed at assessing the participants' knowledge of food safety and hygiene practices. These questions covered various aspects such as general food safety, frozen and refrigerated foods, raw meat/foods, canned/preserved foods, and FBDs, with a maximum score of 54. The median score of 36 was designated as the cutoff point for determining adequate knowledge.

The third section of the questionnaire comprised eight statements regarding the participants' attitudes towards food safety practices. Participants were asked to indicate their level of agreement or disagreement with these statements. The maximum possible score in this section was 32, with the median attitude score of 25 chosen as the cutoff point.

A pilot study involving five participants was conducted to assess the questionnaire's reliability. Cronbach's alpha coefficient yielded a value of 0.8, indicating satisfactory internal consistency among the questionnaire items. Subsequently, the questionnaire was distributed in both printed and online formats, with participants selected through convenient sampling.

Statistical analysis

Data analysis was performed using IBM SPSS Statistics for Windows, Version 26.0 (Released 2019; IBM Corp., Armonk, New York, United States), employing both descriptive and inferential statistics. The chi-squared test was applied to examine the association between variables, and significant associations were further analyzed using logistic regression. Statistical significance was set at p<0.05.

## Results

Of the 402 participants, 127 (32.1%) were male and 275 (67.9%) were female. Regarding age distribution, 220 (54.7%) were below 30 years old, while 182 (45.3%) were aged 30 years or older. Most participants were from the Southeast Asia region 222 (55.4%), followed by the Eastern Mediterranean region 134 (33.4%). Regarding occupation, the largest group consisted of students (186, 47%), followed by healthcare workers 87 (21.5%). Regarding marital status, nearly half of the participants were married 192 (48%). Only 106 (26.36%) had received food safety training, of whom 46 (43.3%) reported attending such training within the last two years (Table 1).

**Table 1:** Distribution of sociodemographic characteristics of the participants (n=402)

Variable		f	%
Gender	Male	127	32.1
	Female	275	67.9
Age (years)	<30 years	220	54.7
	≥30 years	182	45.3
Nationality	African region	19	4.7
	Region of the Americas	10	2.5
	Southeast Asia region	222	55.4
	European region	5	1.2
	Eastern Mediterranean region	134	33.4
	Western Pacific region	12	2.7
Occupation	Student	186	47
	Healthcare worker	87	21.5
	Non-healthcare worker	99	25
	Unemployed	30	6.6
Marital status	Married	192	48
	Unmarried	210	52
Living arrangement	Alone	36	9
	2-5 people	343	85.3
	>5 people	23	5.7

Less than half of the participants 187 (46.51%) demonstrated adequate knowledge of food safety and hygiene practices. Participants exhibited the highest level of awareness regarding food safety practices related to raw foods, achieving an average score of 64.02%. They showed a similar level of understanding of general food safety, FBDs, and canned foods, with average scores of 62.68%, 54.83%, and 40.79%, respectively. In the frozen food section, the average score attained was 47.8%. Table 2 provides detailed information on participants' knowledge regarding food safety and hygiene.

**Table 2:** Knowledge of food safety and hygiene (n=402) FBDs: foodborne diseases

Question (correct response)	f	%
General food safety		
Wiping hands on a dish/kitchen towel increases the risk of food contamination: (True)	290	72.1
Using the same sink for washing dishes and hands increases the risk of food contamination: (True)	253	62.9
Long and/or painted nails increase the risk of food contamination: (True)	339	84.3
Accessories such as rings and bracelets cannot be worn while preparing or handling food: (True)	228	56.7
Food that is no longer edible shows changes in color, odor, and/or taste: (True)	26	6.4
Cuts and wounds must be covered before handling or preparing food: (True)	377	93.7
Frozen and refrigerated foods		
The safe operating temperature range for a refrigerator is: 1-5℃	198	49.2
Most bacteria are destroyed by refrigeration or freezing: (True)	174	43.2
The correct method for thawing frozen meat is: microwave	125	31.1
Frozen meat can be left to thaw overnight at room temperature: (False)	241	59.9
Reheating food increases the risk of food contamination: (True)	224	55.7
Raw meat/foods		
Using gloves while handling raw meat can reduce the risk of food contamination: (True)	333	82.8
Raw and cooked meat must be stored separately: (True)	360	89.5
Cutting boards used for raw meat can be used for other foods without being washed: (False)	354	88.1
Which of the following is the best method for measuring the doneness of meat? (Kitchen thermometer)	138	34.3
What is the minimum internal temperature required to ensure safety while cooking chicken and other poultry? (165℉)	102	25.4
Canned/preserved foods		
Canned/preserved foods are not safe from bacteria: (True)	210	52.2
Consuming canned food is a safer alternative to foods preserved by other means (brine, oil, etc.): (False)	69	17.2
Knows signs of spoiled canned food (bulging and broken seal, corrosion, oozing, bubbles in the jar, food cloudy): (True)	213	52.98
FBDs		
FBDs are caused by bacteria, viruses, and fungi: (All of them)	256	63.7
A person with blood-borne diseases (e.g., HIV, hepatitis B, hepatitis C) can prepare food for others safely: (True)	150	37.3
Knows the route of transmission of FBDs: (Contaminated hands, infected food handlers, dirty utensils, contaminated food/water)	260	64.67
Which age group is at high risk for FBDs: (Older adults)	163	40.54
Knows the common symptoms of FBDs: (Nausea, vomiting, diarrhea)	303	75.3
Knows the common causative organism: (*Escherichia coli*, *Salmonella*, *Staphylococcus aureus*)	191	47.5

The data presented in Table 3 outline the association between various demographic factors and levels of knowledge concerning food safety. Males 79 (62.2%) demonstrated inadequate knowledge compared to females 48 (37.8%); in contrast, females showed a more balanced distribution, with 136 (49.4%) having inadequate knowledge and 139 (50.6%) possessing adequate knowledge (p=0.017). Regarding age, individuals aged <30 years exhibited inadequate knowledge at 125 (56.8%), while those aged ≥30 years showed inadequate knowledge at 90 (49.5%) (p=0.117). Analysis by nationality did not reveal statistically significant differences in knowledge levels across various regions. However, occupation notably influenced knowledge levels, with students demonstrating inadequate knowledge 104 (55.9%) and adequate knowledge 82 (44.1%). Healthcare workers displayed a contrasting pattern, with 31 (35.6%) having inadequate knowledge and 56 (64.4%) having adequate knowledge (p=0.003).

**Table 3:** Association between knowledge and sociodemographic characteristics (n=402)

Demographic		Knowledge		p-value
		Inadequate knowledge (n=215)	Adequate knowledge (n=187)	
Gender	Male (127)	79 (62.2)	48 (37.8)	0.017
	Female (275)	136 (49.4)	139 (50.6)	
Age (years)	<30 years (220)	125 (56.8)	95 (43.2)	0.117
	≥30 years (182)	90 (49.5)	92 (50.5)	
Nationality	African region (19)	9 (47.4)	10 (52.6)	0.541
	Region of the Americas (10)	4 (40)	6 (60)	
	Southeast Asia region (222)	117 (52.7)	105 (47.3)	
	European region (5)	3 (60)	2 (40)	
	Eastern Mediterranean region (134)	75 (56)	59 (44)	
	Western Pacific region (12)	7 (58.3)	5 (41.6)	
Occupation	Student (186)	104 (55.9)	82 (44.1)	0.003
	Healthcare worker (87)	31 (35.6)	56 (64.4)	
	Non-healthcare worker (99)	61 (61.6)	38 (38.4)	
	Unemployed (30)	19 (63.3)	11 (36.6)	
Marital status	Married (192)	92 (47.9)	100 (52.1)	0.031
	Unmarried (210)	123 (58.5)	87 (41.5)	
Living arrangement	Alone (36)	26 (72.2)	10 (27.8)	0.044
	2-5 people (243)	125 (51.4)	118 (48.6)	
	>5 people (123)	64 (52.1)	59 (47.9)	

More than half, 226 (56.2%), of the participants had a positive attitude towards food safety and hygiene practices. Overall, most of the population showed favorable responses in this section. The majority 304 (75.6%) of the respondents strongly agreed that food safety takes priority over taste, and 295 (73.4%) indicated the importance of making food safety information more easily accessible. The majority 300 (74.7%) believed hand sanitizers are not a complete replacement for handwashing during food preparation and handling. Two hundred and seventeen (54%) strongly agreed that gloves, masks, and hairnets are necessary during food preparation, and 220 (54.7%) concurred that individuals suffering from diarrhea, flu, or sore throat should not be involved in food preparation. Mostly, 183 (45.5%) believed food left outside for an extended period should be thrown away immediately; however, only a total of 145 (36%) disagreed with the idea that food should only be thrown away once it shows visible signs of spoilage. The population had poor attitudes regarding the use of kitchen thermometers, with only 31 (7.7%) strongly believing that they are necessary for measuring the doneness of meat (Table 4).

**Table 4:** Attitude towards food safety and hygiene practices

Attitude	Strongly agree	Agree	Disagree	Strongly disagree
Food safety is more important than taste	304 (75.6)	87 (21.7)	5 (1.2)	6 (1.5)
Information about proper food safety and preparation methods should be more accessible to the public	295 (73.4)	95 (23.6)	7 (1.7)	5 (1.2)
Using hand sanitizers can completely replace handwashing during food preparation and handling	42 (10.4)	60 (14.9)	185 (46.1)	115 (28.6)
During food preparation, gloves, masks, and hairnets must always be worn	217 (54)	114 (28.3)	31 (7.7)	40 (9.9)
Food that has been left outside for a long time should be thrown away immediately	183 (45.52)	174 (43.28)	35 (8.7)	10 (2.5)
Food should only be thrown away once it shows visible signs of spoilage	121 (30.1)	136 (33.7)	112 (27.8)	33 (8.2)
Kitchen thermometers are not necessary for measuring the doneness of meat	45 (11.2)	198 (49.25)	128 (31.84)	31 (7.7)
A person having diarrhea, flu, or sore throat should not prepare food for others	220 (54.7)	133 (33.1)	42 (10.4)	7 (1.7)

Table 5 summarizes the association between sociodemographic characteristics and attitudes towards food safety. The groups reported to have more positive attitudes are women 157 (57.1%), individuals above 30 years of age 110 (50.5%), those from the Western Pacific region 8 (66.7%), individuals working in healthcare or non-healthcare 54 (62.1%) and 61 (61.6%) respectively, and married individuals 117 (60.9%). Despite these variations among different sociodemographic groups, no significant correlations were found.

**Table 5:** Association between attitude and sociodemographic characteristics

Demographic		Attitude		p-value
		Negative attitude (n=175)	Positive attitude (n=227)	
Gender	Male (127)	57 (44.9)	70 (55.1)	0.385
	Female (275)	118 (42.9)	157 (57.1)	
Age (years)	<30 years (220)	103 (46.8)	1117 (53.2)	0.106
	≥30 years (182)	72 (39.5)	110 (60.5)	
Nationality	African region (19)	9 (47.4)	10 (52.6)	0.542
	Region of the Americas (10)	4 (40)	6 (60)	
	Southeast Asia region (222)	99 (44.6)	123 (55.4)	
	European region (5)	2 (40)	3 (60)	
	Eastern Mediterranean region (134)	57 (42.5)	77 (57.5)	
	Western Pacific region (12)	4 (33.3)	8 (66.7)	
Occupation	Student (186)	85 (45.7)	101 (54.3)	0.231
	Healthcare worker (87)	33 (37.9)	54 (62.1)	
	Non-healthcare worker (99)	38 (38.4)	61 (61.6)	
	Unemployed (30)	15 (50)	15 (50)	
Marital status	Married (192)	75 (39.1)	117 (60.9)	0.095
	Unmarried (210)	100 (47.6)	110 (52.4)	

Table 6 describes the association between knowledge and attitude towards food safety and hygiene practices. A strongly significant correlation (p<0.001) was found between the two variables; individuals with adequate knowledge were more likely to display positive attitudes (67.9%) than individuals with inadequate knowledge (46.1%).

**Table 6:** Association between knowledge and attitude

Knowledge	Attitude		p-value
	Negative attitude	Positive attitude	
Inadequate knowledge	53.9%	46.1%	<0.001
Adequate knowledge	32.1%	67.9%	

Table 7 describes the significant sociodemographic factors associated with knowledge of food safety and hygiene practices. Women were 1.68 times more likely to have adequate knowledge than men (CI=1.09-2.59). Healthcare workers were 2.33 times more likely to have adequate knowledge than students (CI=1.37-3.95). Non-healthcare workers or unemployed individuals were not found to have a statistically significant increase or decrease in adequate knowledge compared to students. Married individuals were 1.49 times more likely to display adequate knowledge than unmarried individuals (CI=0.99-2.21). Individuals living with 2-5 people were 2.47 times more likely to display adequate knowledge than those living alone (CI=1.15-5.27), while those who live with >5 people were not significantly more or less likely to have adequate knowledge. After adjusting for other variables, none of the above factors were found to be significant.

**Table 7:** Factors associated with knowledge of food safety and hygiene practices

Knowledge	OR (95% CI)	p-value
Gender (female)	1.68 (1.09, 2.59)	0.018
Occupation (healthcare)	2.33 (1.37, 3.95)	0.002
Marital status (married)	1.49 (0.99, 2.21)	0.050
Living (2-5 people)	2.47 (1.15, 527)	0.020

## Discussion

Due to its detrimental effects on public health and the economy, food safety is considered a top priority by consumers, food control agencies, and the food services industry. Earlier studies have identified improper food handling practices as the primary cause of foodborne illnesses in restaurants [10,11]. According to the current study, adults in the UAE demonstrated a good overall level of food safety knowledge and attitude. Specifically, participants exhibited the highest level of awareness regarding "food safety practices related to raw foods," "understanding of general food safety," "foodborne diseases (FBDs)," and "canned foods," while a fair level of awareness was demonstrated about "food safety and hygiene practices" and "frozen and refrigerated foods." However, the overall percentage of food safety knowledge (46.1%) in this study was lower than the percentages reported in earlier studies conducted in three European Union (EU) countries (70.5%) [12] and other regions such as Portugal (56.5%) [13], Kuwait (70%) [14], and the UAE (70%) [15]. Additionally, a higher score for the "food safety and personal hygiene" aspect (80%) was reported in the Kingdom of Saudi Arabia (KSA) [16] compared to our study. This variation could be attributed to differences in sociodemographic characteristics, study design, and the study period. Several factors, including gender, occupation, marital status, and living arrangements, were significantly correlated with knowledge.

Women scored higher in the knowledge section of the questionnaire (50.6%), likely because they play a larger role in food preparation, particularly for their families. This aligns with studies in Greece [6], Pakistan [9], Italy [17], and Maine [18]. However, studies in Brazil [4], Lebanon [8], and Palestine [19] found no significant correlation.

Healthcare workers had greater knowledge of food safety and hygiene practices than other occupational groups, likely due to the training involved in their work. Unemployed individuals, including housewives, had the second-highest knowledge score, presumably due to their involvement in food preparation for their families [19].

Age was observed to affect participants' knowledge, but the association was not significant (p>0.05), a finding reflected in other studies [4,19,20]. Training was not found to be significantly correlated with food safety knowledge, contrary to many studies [20-22]. It's possible that training is not effective in promoting the retention of food safety knowledge, especially if it is lengthy, is not repeated within 2-5 years, lacks a balance of theoretical and practical knowledge, or is not accompanied by continuous support and supervision to encourage positive attitudes [23,24].

Respondents who were informed about food safety methods through their occupation showed significantly more positive attitudes towards food safety (p=0.041). Continual exposure to food safety-related information has been found to significantly influence the transfer of knowledge to both attitude and practice [25].

There was no significant association between attitude and food safety training. The lack of repetitive training and infrequent support and supervision may contribute to this finding [23]. In general, the population reported favorable attitudes towards various aspects of food safety. The majority claimed to prioritize food safety over taste was supported by other studies [26,27].

A strong and significant correlation between knowledge and attitude was observed in this study, aligning with findings from earlier studies [27-29]. Similarly, the absence of a significant correlation between attitude and practice was observed in certain populations [30,31]. The lack of a significant correlation between knowledge or attitude and practice is likely due to social desirability and self-assessment biases in self-reported practices.

Limitations

Our study encountered several methodological limitations. Potential response biases, including social desirability and recall, could have inflated estimates. As a cross-sectional design, inferring causality remains challenging. A significant limitation was that we only assessed self-reported practices, which likely contributed to the observed discrepancies between reported practices and other factors.

## Conclusions

The findings highlight a notable deficit in food safety knowledge among adults in the UAE, underscoring the need to enhance awareness to mitigate the impact of foodborne illnesses on healthcare. With only 46.1% of participants demonstrating adequate knowledge and 56.2% exhibiting favorable attitudes towards food safety, there exists a significant correlation between stronger knowledge and a more positive attitude. These insights are crucial for food control authorities, food service management, and food safety trainers, informing targeted efforts to address knowledge gaps and promote safer food practices.

## Appendices

Questionnaire

Section 1: Sociodemographic Details

Gender:                       M                     F                      Prefer not to say

Age: _________

Nationality: ____________

Occupation: ___________                 If you are a student, please state your program _________

Marital Status:             Married            Unmarried       Prefer not to say

How many people live in your residence?

I live alone                                           2-5 people                                           >5 people

Have you ever received formal training for food safety and hygienic practices?

Yes                                                      No

If you answered yes to the previous question, how recently did you attend training for food safety?

<2 years ago                                     2-4 years ago                                      >4 years ago

Section 2: Knowledge of Food Safety Practices

Please circle the correct answer.

Section 2a: General food safety

Wiping hands on a dish/kitchen towel increases the risk of food contamination.

True                                                     False                                                   Unsure

Using the same sink for washing dishes and hands increases the risk of food contamination.

True                                                     False                                                   Unsure

Long and/or painted nails increase the risk of food contamination.

True                                                     False                                                   Unsure

Accessories such as rings and bracelets can be worn while preparing or handling food.

True                                                     False                                                   Unsure

Food that is no longer edible always shows changes in color, odor, and/or taste.

True                                                     False                                                   Unsure

Cuts and wounds must be covered before handling or preparing food.

True                                                     False                                                   Unsure

Section 2b: Frozen and refrigerated foods

The safe operating temperature range for a refrigerator is:

1-5℃                                                    6-10℃                                                  11-15℃    

Most bacteria are destroyed by refrigeration or freezing.

True                                                     False                                                   Unsure

The correct method for thawing frozen meat is:

In the refrigerator        At room temperature              In the microwave                    Unsure

Frozen meat can be left to thaw overnight at room temperature.

True                                                     False                                                   Unsure

Reheating food increases the risk of food contamination.

True                                                     False                                                   Unsure

Section 2c: Raw meat/foods

Using gloves while handling raw meat can reduce the risk of food contamination.

True                                                     False                                                   Unsure

Raw and cooked meat must be stored separately.

True                                                     False                                                   Unsure

Cutting boards used for raw meat can be used for other foods without being washed.

True                                                     False                                                   Unsure

Which of the following is the best method for measuring the doneness of meat?

Kitchen thermometer              Taste               Smell              Appearance                 Unsure

What is the minimum internal temperature required to ensure safety while cooking chicken and other poultry?

100℉ (38℃)                145℉ (63℃)                165℉ (75℃)                200℉ (93℃)

Section 2d: Canned/preserved foods

Which of the following are signs of spoiled canned food? (You may select more than one answer)

❏     A bulging can or lid or a broken seal

❏     A can or lid that shows signs of corrosion

❏     Food that has oozed or seeped under the jar's lid

❏     Gassiness (bubbles in the jar/can)

❏     Food appears cloudy, mushy, or moldy

❏     Unsure

Canned/preserved foods are safe from bacteria.

True                                                     False                                                   Unsure

Consuming canned food is a safer alternative than foods preserved by other means (brine, oil, etc.).

True                                                     False                                                   Unsure

Section 2e: Foodborne diseases (FBDs)

FBDs are caused by:

Bacteria           Viruses            Fungi              All of the above                  Unsure

Which of the following are routes of transmission for FBDs? (You may select more than one answer)

❏     Contaminated hands

❏     Infected food handlers           

❏     Dirty utensils  

❏     Disease vectors         

❏     Contaminated food/water

❏     Dirty work environment

❏     Unsure

Which of the following groups are at lower risk for FBDs? (You may select more than one answer)

❏     Older adults

❏     Teenagers and young adults

❏     Pregnant women

❏     Infants and young children

❏     People with chronic diseases

❏     Unsure

Which of the following are common symptoms of FBDs? (You may select more than one answer)

❏     Nausea

❏     Vomiting

❏     Watery/bloody diarrhea

❏     Abdominal pain/cramps

❏     Fever

❏     Headaches

❏     Dizziness

❏     Unsure

Which of the following organisms commonly cause FBDs? (You may select more than one answer)

❏     *Campylobacter*

❏     *Clostridium perfringens*

❏     *Escherichia coli* (*E. coli*)

❏     *Listeria*

❏     *Clostridium botulinum*

❏     *Salmonella*

❏     Unsure/I don't know

A person with blood-borne diseases (e.g., HIV, hepatitis B, hepatitis C) can prepare food for others safely.

True                                                     False                                                   Unsure

Section 3: Attitude Towards Food Safety Methods

**Table 8:** Attitude towards food safety methods

Statement	Strongly agree	Agree	Disagree	Strongly disagree
Food safety is more important than taste.				
Information about proper food safety and preservation methods should be more accessible to the public.				
A person having diarrhea, flu, or sore throat should not prepare food for others.				
During food preparation, gloves, masks, and hairnets must always be worn.				
Using hand sanitizers can completely replace handwashing during food preparation and handling.				
Food that has been left outside for a long time should be thrown away immediately.				
Food should only be thrown away once it shows visible signs of spoilage.				
Kitchen thermometers are not necessary for measuring the doneness of meat.				
